# Hyper-activated PI3K-δ in immunodeficiency

**DOI:** 10.18632/oncotarget.4884

**Published:** 2015-07-17

**Authors:** Lucie Heurtier, Marie-Céline Deau, Sven Kracker

**Affiliations:** Université Paris Descartes, Sorbonne Paris Cité, Imagine Institute, INSERM UMR 1163, Human Lymphohematopoiesis Laboratory, Paris, France

The class I phosphoinositide 3-kinases (PI3Ks) are especially involved in the cell response to extracellular signals and convert phosphatidylinositol-(4,5)-bisphosphate (PIP2) into phosphatidylinositol-(3,4,5)-triphosphate (PIP3), an important intracellular “second messenger”, activating many different intracellular enzymes, including Akt also known as protein-kinase B. Class IA PI3Ks are heterodimeric molecules consisting of a catalytic subunit (p110α, p110β or p110δ) and a regulatory subunit (p85α, p85β, p85γ, p50α or p55α). Each of the catalytic subunits can bind to any of the regulatory subunits. The p110δ catalytic subunit is expressed predominantly in leukocytes, whereas the other subunits present with an ubiquitously expression pattern.

Class IA PI3Ks activation in lymphocytes occurs downstream of the antigen receptor (B or T cell receptor) and co-stimulatory molecules. Crosslinking of antigen receptors leads to phosphorylation of tyrosine residues, which then recruits class IA PI3Ks [1].

A homozygous nonsense mutation in *PIK3R1* causing loss of p85α was identified in an immunodeficient patient presenting with agammaglobulinemia [2]. More recently heterozygous gain-of-function mutations in the *PIK3CD* gene encoding p110δ have been described (N334K, C416R, E525K and E1021K with the majority of cases carrying E1021K) as a cause for primary immunodeficiency [3-5]. The disease was called activated PI3K-δ syndrome (APDS) [3]. Frequent symptoms were recurrent respiratory infections since childhood, bronchiectasis, lymphoproliferation, adenopathy and hypogammaglobulinemia or Hyper-IgM syndrome. Abnormalities of lymphocyte subpopulations were observed in the B (higher frequency of transitional, decrease of mature B) and T lymphocyte compartments (decreased naïve CD4 T and naïve CD8 T and increased frequency of effector memory CD8 T cells) indicating a combined immunodeficiency. APDS appears to predispose to B cell lymphomagenesis especially diffuse large B cell lymphomas and Hodgkin lymphoma. A combination of aberrant immune surveillance due to disturbed T cell function and B cell intrinsic defects could be responsible for the increased B cell lymphoma frequency.

We identified by using the whole-exome sequencing approach a novel cause for hyper-activated PI3K-δ signaling: splice site mutations in *PIK3R1* [6]. *PIK3R1* encodes the most abundantly expressed regulatory subunit p85α and two additional isoforms (p55α and p50α), the production of which is regulated by two distinct promoters and an alternative exon 1. These mutations located at the splice donor site of the coding exon 10 lead to exon skipping and affect the mRNAs of all *PIK3R1* encoded isoforms. Splicing from exon 9 to exon 11 occurs in frame thus these mutations lead to a shortened p85α protein which was detected in total protein extracts from patient derived T cell blasts, although less abundant than the wild-type p85α protein. P110δ abundance in the T cell blasts were similar to those observed in controls. The coding exon 10 of the *PIK3R1* gene encodes part of an alpha helix known to be involved in the p85α/p110 interaction. Co-immunoprecipitation experiments with transiently expressed tagged-p85α and tagged-p110δ demonstrated that the shortened p85α protein interacted with p110δ as efficiently as the wild-type p85α protein (own unpublished observations, [7]). Phosphorylated Akt (at Ser473 and Thr308) was present in patients IL2-derived T cell blasts without further activation in contrast to control cells demonstrating hyper-activation of the PI3K signaling. The comparison of phosphorylated Akt at Ser473 in murine fibroblast ectopically expressing either the shortened or wild-type p85α protein confirmed that the shortened p85α protein is responsible for the hyperactivated PI3K signaling. As observed for T cells from APDS patients, an increased ‘activation-induced-celldeath’ (AICD) was detected in patients’ derived IL2 T cell blasts. Pre-incubation of cells with the PI3Kδ inhibitor IC87114 reduced the increased AICD in patient cells. In addition, patients T cell blasts treated with the PI3Kδ-inhibitor presented with normal Akt phosphorylation indicating that p110δ mediated signaling is responsible for the hyper-activated phenotype. Thus we named this new immunodeficiency “activated PI3K-δ syndrome 2” (APDS2) [6]. Treatment of one APDS patient with rapamycin resulted in the reduction in hepatosplenomegaly and lymphoadenopathy and an increase in the frequency of naive T cells [4]. Based on this report, some APDS1 and APDS2 patients are currently treated with rapamycin. Our results suggest that a p110δ specific inhibitor could be a more attractive treatment for this disease, given that it targets the specific pathological protein.
